# The Hospital Nursing Supplement

**Published:** 1892-11-26

**Authors:** 


### "The Hospital" Nursing Mirror.

BEING THE EXTRA NURSING SUPPLEMENT OF "THE HOSPITAL" NEWSPAPER.

[Contributions for this Supplement should be addressed to the Editor, THE HOSPITAL, 140, Strand, London, W.C., and should have the word "Nursing" plainly written in left-hand top corner of the envelope.]

### En Passant.

METROPOLITAN ASYLUMS BOARD.—This Board decided this past week that the gratuities to be paid to members of the subordinate staff at the infectious hospitals of the Board who may be granted leave of absence when recovering from infectious disorders contracted in the performance of their duties be as follows: To superintendent nurses and nurses, 30s. per week; assistant nurses, 25s. per week; and any other member of the subordinate staff, 20s. per week.

ST. JOHN'S MATERNITY HOME.—We much regret to hear that St. John's House, Norfolk Street, Strand, has given up its Maternity Home at Battersea, and the good work carried on there solely for want of funds. The Home, which has flourished in all other ways under the able management of the Sister Superior, one of the All Saints' Sisters, has not been well supported pecuniarily by the public, and so the Council reluctantly decided that it was not justified in continuing the necessarily heavy expenditure any longer. During the time the Home has been in existence it has passed many candidates at the London Obstetrical Society's examination, success being the rule, and failure the exception. There has been no failure since October, 1890, and since that time over 40 pupils have gained the diploma of the Society.

HOME FOR NURSES, CAMBRIDGE.—We hear there is much sorrow at the Home in Fitzwilliam Street at Miss Young's departure at Christmas time, as she has done so much to promote happiness and kindliness there, and, amongst other improvements, has granted percentage on each nurse's earnings. The nurses have given a writing table to Miss Young as a memento of the esteem in which they all hold her. Miss Young is not leaving Cambridge, but she is about to start "daily nursing" for the middle classes, that is for those who either cannot arrange to employ trained nurses by the week, but would be glad of the skilled assistance which could be given in one or two visits a day. Miss Curtis, who trained at St. Bartholomew's for three years, and who is now in charge of a ward there, will succeed Miss Young.

THE WANT OF MONEY.—There is a certain sameness in the perpetual cry we hear of the urgent need of funds for everything and everybody just now, but it is sadly true that many of our best institutions are languishing for lack of wherewithal. Bolingbroke House Paying Hospital, Wandsworth, is one among the number which counts itself to be very hard up, and as we know of numberless cases which have met with the greatest kindness within its doors, we record the fact with much regret. The principle upon which it has been carried on differs from that of other paying nursing homes, in that the scale of payment is so arranged that each patient pays just so much as he can reasonably afford, and it was originally hoped that wealthier patients would be able to compensate for any loss incurred in the liberal treatment of poorer brethren. Such, however, is not the case, and we do hope that those who have reaped the benefits of this hospital, either directly to themselves or indirectly to their friends and relations, will try to raise up new friends and relieve the committee in its present need.

BELFAST NURSES.—Miss Martin, who became District Matron in this town in October, 1891, has been obliged, for private reasons, to resign her post, and Miss Moore, late of Ventnor Consumption Hospital, took her place on the first of this month. Nurse M'Comb, who has been on the staff for thirteen years, was obliged to retire in September on account of ill-health.

KINGSTON BOARD OF GUARDIANS.—The Inspector, when at Kingston lately, said that he wished to draw the attention of the committee to several points, and one was the necessity of appointing a night nurse. He quoted many cases where the Local Government Board have got night nurses appointed. The Chairman pointed out that nine-tenths of the sick in the house were chronic cases, and not acute ones requiring urgent medical treatment, but only ordinary attention. What an extraordinary fallacy it is that the chronic sufferer should be deemed an object of so small importance! The fact that night and early morning are the times at which the greater averages of deaths occur, and the loneliness and dreariness of the night, well remembered by any of us who have been ill, should be sufficient reasons for appointing night nurses, whose presence is of as great or greater importance during the night than the daytime.

NURSING IN INDIA.—A year has now elapsed since the Mofussil Nurses' Association was started, and the scheme seems to be progressing. Having no home of its own, the Association thereby saves considerable cost in administration. The three main objects of the society are to reserve, for the benefit of the subscribers, the services of certain trained nurses; to assist those who need the services of trained nurses, and cannot pay for them by contributions towards the fees; and to interest up-country Europeans in a scheme which will strengthen and assist existing agencies for nursing. With this latter object in view the work of the Association should be of value as being subsidiary to the two central nurses' homes attached to the hospitals in Bombay and Poona respectively.

THE PINNACLE OF PERFECTION.—There is certainly no lack of pride of profession among either nurses or asylum attendants, and the least adverse comment rouses numbers to arms, and emphatic denials that anything but what is perfect pertains to either calling shower in upon the unlucky commentator. This is good to a certain point, but after this point it is well to look round and see whether we are sure that we are without the least flaw in our system. There are bad nurses and bad attendants, and when they hear or see critical remarks they have an uncomfortable sensation that there is some truth in certain statements, and that in other words the "cap fits," but it is quite unnecessary for those who are giving their life's earnest work up to best endeavours to worry themselves at all; we none of us like to feel that there are second-rate workers in our ranks, but, unfortunately, such is the case, and it is a great mistake to appear indignantly unconscious of this fact. If we are all going to set ourselves upon a pinnacle of perfection, how are we ever to eliminate the bad; and there is the danger that from very conceit we shall scorn to look around and so gradually lower our standard of efficiency.

### Lectures for Asylum Attendants.

By William Harding, M.B.

### IX.—EPILEPTICS.

One of the things which make the greatest impression upon a beginner in asylum work is the matter-of-fact way in which "fits" are treated. It is part of a nurse's duty to record the number of fits that patients may have during her period on duty, and also to make a report should there be anything unusual about any of the seizures. She ought, therefore, to be familiar with the forms in which epilepsy may show itself. The ordinary fit is a convulsive seizure, accompanied by loss of consciousness, and frequently preceded by a characteristic cry. The convulsion is the marked feature in the great majority of our cases, but there are some who do not show this prominent symptom, and such cases should be carefully watched. In these individuals the seizures are manifested merely by a loss of consciousness sometimes so temporary as to be unnoticed by any but one who knows the nature of such a symptom and is on the lookout for it. The patient may simply appear dazed for a few minutes, and be unconscious of what is going on about her; she may look as if she had a slight fainting attack; or she may perform some ordinary or out-of-the-way action without being aware of what she is doing. These are as surely indications of the disease as is the ordinary fit, and should be looked for and noted. Undoubtedly there are patients who have seizures of this kind which pass unnoticed for a long time, but would not do so if the nurses were aware of the full significance and importance of such things. The nurse will find that the fits of different individuals vary much in character. She will also find that there are great variations in the sensations of which patients complain, as well as in their behaviour immediately before having a fit. She should make herself familiar with these warning symptoms, and when they appear she should try to put the patient in a situation such that she is not likely to injure herself when the fit occurs. Of course there are many who have the seizure without any such warning, and often disfigure themselves in the severe falls they have. The danger of an epileptic falling near the open fire, the stove, or the steam coil is apparent. There is also danger in the going up and down stairs, and those patients who are likely to have fits should be closely looked after on such occasions. At night epileptics must be kept under constant supervision, with the face uncovered and in the nurses' view. There is always a danger that the patient may have a fit without any cry to attract attention to her, and in turning over on her face may suffocate herself. So far as attention during the night is concerned, the pauper lunatic is the pampered child of civilisation. How many sufferers from epilepsy, even amongst the highest classes, are watched day and night as she is? No patient suffering from epilepsy should be permitted to mount on step-ladders to clean windows, nor, indeed, to get into any position where they would be likely to injure themselves in falling in a fit.

When a fit does occur the patient should be laid down on the floor or on a couch and a cushion placed under her head. No more force should be used, nor, indeed, any interference other than is sufficient to prevent the patient injuring herself. The clothing about the neck and the chest, if necessary, should be loosened. It is not likely in our asylum patients that there will be any tight lacing to undo: that is one of the insane habits which are dropped at the asylum doors. When the convulsive stage is over the patient should be left quietly on a couch to recover.

When the epileptic is "having her fits" she is generally much disturbed in mind, and is a great strain upon the nurse's patience and temper. The usual effect of epilepsy is to cause the mind to become gradually weaker until at last a stage of nearly complete dementia is reached. Some of the more intelligent in their peri ds of calm between the fits are grateful for what is done for them and anxious to assist the nurses. Such individuals are, as a rule, the best workers amongst the lunatics. As a class, however, the epileptics are bad patients. They often display deep religious feeling, and at the same time are untruthful, given to stealing, prone to making false charges, and are always ready to quarrel. With them the blow generally comes before the word. A fancied insult is frequently the only reason assigned for a savage onslaught upon an unoffending and harmless bystander. If in passing through the wards of an asylum you see a man or woman intently searching the Scriptures, you may with safety, in five cases out of six, risk the statement that the individual is an epileptic. If spoken to, they will very probably make complaints of ill-usage which are utterly unfounded, but which they will back up with fervent appeals to the Deity to witness the truth of what they say. They will also show some marks, received in a fit, and accuse some nurse or patient near of having inflicted them. These are the individuals, and especially if hysterical, who bring the great majority of the charges of cruelty against the nurses. This is probably due to a morbid longing for sympathy: a warped moral sense, and in a great measure to the stiffness and aches after a fit being attributed by the sufferer to ill-usage from those about her. As the nurses have frequently to exercise some control over the patient's movements during or immediately after a fit, and she finds them by her when her glimmering consciousness returns, she naturally refers her sufferings to their agency.

When excited, the epileptic is difficult to manage. She is suspicious, impulsive, abusive; quickly roused to violence, and in the heat of passion will do deeds of which she is perfectly ignorant when her wild fury has passed away. Many of our criminal lunatics belong to this class. The homicidal impulse may be quite irresistible, and murder may be committed without the patient being afterwards in the least aware of what has happened. It is curious, also, how often we find the propensity to steal, and afterwards conceal what has been stolen. They will, even if caught in the act, stoutly deny the possibility of their ever dreaming of doing such a thing. A nurse should always be extremely careful in handling an epileptic. When irritable they will resent a hand being laid on their arm, even in the most friendly way, and will impulsively strike out. When in that mood, coaxing methods should always be tried and abrupt dealings avoided. A nurse should never engage in a single handed struggle with an epileptic. In such circumstances there is great danger of the patient or nurse being injured. The former's powers of self-control are much weakened, and when in a state of maniacal excitement she is utterly lost to all sense of reason. If the attention of the epileptic be fixed upon any object it is not easily diverted, and the persistent way in which she will with stammering utterance repeat again and again the same pointless question is very trying. During their periods of excitement such cases are best treated by being put to bed in a room by themselves until they are calmer. Such a course is much the best both for themselves and for the other occupants of the wards. It must, of course, be always borne in mind that they must be kept under continuous observation lest suffocation in a fit should occur.

The condition of the bowels should be remembered. Constipation is common, and the nurse should report if any under her care are suffering in that way. The free unloading of any epileptic's intestine will in some cases do more towards lessening excitement than any amount of sedatives.

(*To be continued.*)

### The Registration of Nurses and the R.B.N.A.

### APPLICATION TO THE PRIVY COUNCIL.

A Special Committee of the Privy Council sat on Monday, the 21st inst., in the Council Chamber of the Privy Council at Downing Street, to hear the application of the Royal British Nurses' Association for a Charter of Incorporation. The Committee was composed of the Marquis of Ripon, Viscount Oxenbridge, Lord Hobhouse, and Lord Hannen.

The Royal British Nurses' Association were represented by Sir Horace Davey, Q.C., Mr. Muir Mackenzie, and Mr. W. H. Cross; Sir Richard Webster, Q.C., M.P., and Mr. L. S. Bristowe appeared for the Nightingale and other Nurse Training Schools, Hospitals, and Infirmaries throughout the United Kingdom which were opposed to the registration scheme of the R.B.N.A.

The arguments of counsel were not completed when the Committee rose, and the further hearing of the case was adjourned to Monday, the 28th inst. The matter being, therefore, still *sub judice*, we refrain from making any comments upon the case, and confine ourselves to a bare statement of the facts as set forth in the printed cases lodged on each side by order of the Privy Council, and to a report of the arguments addressed to the Court.

It should, however, be observed that counsel on behalf of the Association disclaimed at the outset any desire to form a register of nurses which should be a conclusive guarantee to the public of their qualifications, or, indeed, to do more than publish a list of names of nurses with the place and length of hospital experience which each possessed, so as to put persons desirous of engaging a nurse in possession of information upon which further inquiry could be based. The opposition has throughout been directed not against a directory of nurses, but against a register purporting to be authoritative.

### Foundation and Objects.

It will be remembered that in the year 1886 The Hospitals' Association appointed a committee, consisting mainly of matrons and sisters of hospitals, to inquire into and report upon the possibility of establishing a general register of nurses.

In consequence of the dissensions which arose in that committee, the majority of its members retired in November, 1887. Those who remained, including the Chairman, Mrs. Bedford Fenwick, and the secretary, Miss Catherine J. Wood, together with certain other members, withdrew from the Hospitals Association, and subsequently, on December 7th, 1887, founded the British Nurses' Association, with H.R.H. Princess Christian as President.

The objects of the new association were stated to be to unite all qualified British nurses in membership of a recognised profession; to provide for their registration on terms satisfactory to physicians and surgeons as evidence of their having received systematic training; to associate them for their mutual help and protection, and for the advancement in every way of their professional work; and with a view to the attainment of those objects to obtain a Royal Charter incorporating the Association, and giving sanction to registers of nurses and midwives. Membership of the Association was confined to nurses, midwives, and medical men.

Its objects were further elucidated in a pamphlet published by its authority, in which the following passage occurred:—

"The British Nurses' Association aims, in the first instance, at bringing about a state of things in which no one will be allowed to call herself a nurse, or, at least, will be regarded as a nurse by the public, unless she has undergone a proper course of hospital training of sufficient length to give her an insight into the management of all kinds of illness, whether medical or surgical."

In its first annual report it was stated that:—

"The British Nurses' Association suggests registration of trained nurses and midwives, as a system which has worked excellently well in protecting the public from imposters in other skilled callings. It has endeavoured to persuade State authorities to undertake the scheme, but one and all have declined to commence so great a work. The Association, therefore, must firmly determine to initiate registration itself. It is supported in this by many of the leaders of the medical profession. It is opposed by some, who make vague assertions of evil, but will give no definite facts in proof of their statements, nor any advice or assistance to the Association in formulating any other remedy, although they do not attempt to deny that the grave evils before mentioned are in existence and on the increase. The General Council, therefore, having asked for this advice and assistance from every one who can give it, finds itself obliged to act on its own initiative. It has decided to open, without further delay, registers of nurses and midwives, and to place on these at once, without payment, the names of members of the Association."

### The Hospitals Association Committee.

The committee of the Hospitals Association above mentioned, having failed to bring their inquiry to a conclusion, a second committee was appointed to pursue the investigation. The second committee sat under the presidency of the late Dr. J. C. Steele, and the report which was in March, 1888, issued under his guidance, contained the results of a most careful and searching examination into the whole question of the registration of nurses.

The committee considered that the first step towards a right knowledge of the subject was to make a formal inquiry into the practice and wishes of the various nurse-training schools in London and throughout the country, with a view of eliciting whether they were desirous of establishing a collective register. To obtain the fullest information on these and other incidental points connected with nurse-training, the committee put itself in communication with 34 establishments in England and Scotland, which profess to educate nurses. To these institutions it addressed a series of questions, of which the first was: Do you consider a general system of registration desirable for qualified nurses?

Of the 34 institutions addressed 24 were associated with medical schools, seven were ordinary medical and surgical hospitals, and three were union infirmaries. Eight of the hospitals communicated with took no notice of the application; one curtly refused to have anything to do with it; six acknowledged receipt, with a promise that the document should receive consideration; 19, comprising 17 large hospitals and two union infirmaries, contributed more or less information.

As regards the answers received to the first question the report stated:—

"Rather less than one-half of the replies are in the affirmative, while the bare majority, comprising, however, the leading and best-known training-schools, desire to be let alone. The grounds of the latter for this opinion are based on the facts that each has established a register of its own to meet its special requirements; that they would lose rather than gain by any system partaking of amalgamation; and that the time has not arrived for a central organisation to be delegated with authority to control the action of the various bodies, commercial and charitable, entrusted with the management of nurses. There is every reason to believe that the following training-schools concur in these opinions: The Nightingale Fund Committee (associated with St. Thomas's Hospital and other institutions), the London Hospital, Guy's Hospital, St. George's Hospital, Westminster Hospital, King's College Hospital, together with the Royal Infirmaries of Manchester, Liverpool, Glasgow, and Edinburgh. It is highly probable also that many of the hospitals which have not yet answered the questions addressed to them have refrained from doing so from an unwillingness to entertain suggestions which would interfere with their local organisation."

The conclusion at which the Committee ultimately arrived was stated in the report as follows:—

"It is manifest to your Committee that the obstacles in the way of organising a general system of registration increase with the knowledge possessed of the working of the various institutions in which nurse-training is carried on. It is equally evident that, unless there is a general unanimity among the training-schools as to the basis on which such a system can be inaugurated, any attempt to form a voluntary register must necessarily prove a failure, while, in the present progressive condition of the nursing question, legislative interference would be attended with disastrous results. The fact that most of the large hospitals, some of which have long been known as pioneers in the movement for the better education of nurses, disapprove of an innovation, which they honestly believe would in a manner dissociate the nurse from her parent school, practically settles the question. A central organisation, possessing a separate jurisdiction, cannot fail more or less to affect the individuality, the healthy rivalry, and the *esprit de corps* which characterise the members of the best training schools, by reducing them to a dead level with others, which are far from possessing the same advantages. It is to the manifest benefit of every hospital to be able to adapt its training, certificating, and registering to meet its own local requirements, and the nature of the duties the nurse may afterwards be called on to perform when she leaves the service of the hospital."

### Intended Application in 1889.

In the year 1889 the British Nurses' Association announced its intention of forthwith applying to her Majesty in Council for a charter of incorporation, and in the Report of the General Council in 1889 it was stated that the charter had been fully drafted, and, except for some verbal alterations which might afterwards be made, was practically prepared.

The draft of a charter stated to be the one referred to in the above report was mentioned in the course of the arguments on the present application, but as it was stated to have been intended as a private document, and not to have been, in fact, adopted by the Association, it was agreed that no reference to it should be made.

In view of this intended application, the authorities of many of the leading hospitals and nurse-training schools published in the *Times* and elsewhere the following memorial of protest:—

"Memorial of Nurse-Training School Authorities.

"We, the undersigned, beg the favour of your insertion of the following statement, which we think it desirable to make, in view of a paragraph which has been published on the subject of the registration of nurses, in which we note with surprise the statement that the main object of the British Nurses' Association is in conformity with a great public want and a widespread professional demand.

"We would wish to point out that those who represent the largest nursing institutions in the metropolis and throughout the country, and who have the most to do with the training and examination of nurses, have not only declined to take part in the Association, but consider that its proposed enrolment of nurses in a common register if carried out would (1) lower the position of the best-trained nurses, (2) be detrimental to the advancement of the teaching of nurses, (3) be disadvantageous to the public, and (4) be injurious to the medical practitioner.

"We hope that a final judgment upon this important matter will be postponed until the views of those who are opposed to the aims of the Association have been expressed and examined. We further consider it our duty to state that if a charter be applied for on the lines stated in the prospectus of the British Nurses' Association we shall feel it to be incumbent upon us to offer thereto all legitimate opposition in our power."

### Application to General Medical Council.

The proposed attempt to obtain a charter was not then proceeded with, but in the month of November, 1889, the Association submitted to the General Medical Council a memorandum proposing (pars. 1 to 4) the formation of a joint register of nurses and midwives to be under the control of the medical and nursing professions, and requesting (par. 5) the advice, support, and assistance of the Council. The scheme proposed by the memorandum was—

"That a registration council should be formed, to which every training school for midwives and nurses in the United Kingdom might be invited to send one member of its medical or nursing staff to represent its views and wishes upon which the Association, as comprising one-fifth of the number of nurses now estimated to be at work, might be represented by the members of its executive committee, and to which other members might be specially added. That this body should meet once or twice a-year, and decide upon all necessary rules and regulations, and be, in short, the governing body of the scheme."

The memorandum was considered by the General Medical Council in connection with certain correspondence which had passed between the President of the Council and Mr. Fell Pease, M.P., in reference to the Bill for the compulsory registration of midwives, which he had introduced into the House of Commons. The Council declined to combine the question of registering nurses with that of registering midwives, the latter being (as Sir John Simon expressed it) a matter forced upon them by Parliament and ripe for legislation, while the former was not. The suggestion of a joint register of nurses and midwives was therefore rejected. The Council at the same time refused to give advice or assistance to the Association as regards the question of registering nurses. The resolution passed on this point was:—

"That in the opinion of the Council it would be much to the advantage of the public and particularly would be of much convenience to the practitioners of medicine and surgery that facilities usable under proper guarantees in all parts of the United Kingdom should be given by Act of Parliament or otherwise for the authoritative certification of competent trained nurses, who when certified should be subject to common rules of discipline; but that it does not appear to be within the province of the Council to propose legislation or other action for the object referred to, nor has the Council had any occasion to consider in detail the means by which the object might best be attained; and that under these circumstances the Council cannot give any opinion on the particular questions asked of it in paragraph five of the Memorandum of the British Nurses' Association."

### Application to Board of Trade.

In the year 1891, the Association applied to the Board of Trade for a licence to be registered as a limited company, without the addition of the word "Limited" to its name. The objects of the proposed company were in the Memorandum and Articles of Association submitted to the Board, stated to be (among others), to form, control, and carry on (1) a register of trained nurses; (2) a register of certificated midwives, and to determine from time to time the test for registration. The hospitals and training schools who had signed the memorial of protest above mentioned, now petitioned the Board of Trade to refuse the license. A petition was also presented, widely signed by many influential members of the medical profession.

Ultimately the Board of Trade refused the application. The grounds of refusal were stated in a letter from the Board dated May 6th, 1891, as follows:—

"After careful consideration of the objects of the Association, and of the representations made in opposition thereto, the Board of Trade are unable to satisfy themselves that the means which the Association propose to adopt are either adequate to carry out their object satisfactorily or so free from objection as to warrant the Board of Trade in the issue of a licence, and under these circumstances they are unable to accede to the application."

A further statement as to the grounds of refusal was, on June 18th, 1891, made by the President of the Board of Trade in Parliament in answer to a question addressed to him by Mr. Paulton, M.P. The President then said:—

"After careful consideration of the application to which the hon. member refers, and also of the representations made to me in opposition to it by bodies aod persons whose interest in hospital nursing is unquestionable, and whose experience entitles them to speak with authority, I was unable to satisfy myself that the means which the Association proposed to adopt were either adequate to carry out its objects satisfactorily or so free from objection as to warrant me in complying with the application to the Board of Trade. I am not aware of any grounds for reconsidering the matter."

The Association's Register.

In June, 1890, the British Nurses' Association having, as stated in the annual report for that year, "done all in its power to persuade others to undertake the scheme (*i.e.*, of registration), found itself compelled to initiate the work single-handed."

It accordingly appointed a Registration Board for the purpose of forming and carrying on a voluntary "Register of Trained Nurses." During a certain period of grace, which expired in the summer of 1890, all applicants for registration were accepted without inquiry.

The Registration Board published its first register in March, 1891, under the name of "The Register of Trained Nurses for 1891." The preface stated that one of the objects for which the Association was founded was the institution of a system of registering nurses analogous to that enforced by law for many years past for medical men, and that the register tacitly asserted two great principles—(1) That the public should be protected from ignorant and untrustworthy women terming themselves trained nurses, and (2) that the control of the nursing profession should be vested solely in professional hands.

A second register was published in the early part of the present year under the name of "The Register of Trained Nurses for 1892." The preface to this register contained statements similar in effect to those in the preface to the previous register, but also stated in addition that the Registration Board had carefully investigated the credentials of every applicant for registration, and had the power of removing from the register the name of any nurse who might prove unworthy of trust, but that it could not answer for the technical knowledge of any registered nurse, or for the nature of the training which she had received, but that every hospital must of necessity be held responsible for the credentials which it had issued, and upon which the Registration Board was compelled to rely.

The last-mentioned disclaimer of responsibility was inserted in consequence of certain criticisms made by Lord Thring in the course of the sitting of the Select Committee of the House of Lords on Metropolitan Hospitals.

From the regulations for registration prescribed by the Association, it appears that applicants are required to produce proof that they have been engaged for three years in work in hospitals or infirmaries, of which not less than twelve months have been spent in a general hospital containing at least forty beds, but that the latter condition, so far as it relates to the number of beds, may be waived under exceptional circumstances by the Registration Board. And it further appears from such regulations that the Registration Board forms its judgments as to the qualification of applicants upon information supplied by the applicant herself, supplemented by confidential information furnished by referees named by the applicant, and that every nurse registered receives a certificate or certified copy of the entry in the register relating to herself, under the seal of the Association.

The right to use the prefix "Royal" was granted to the Association in 1891.

Report of the Lords' Committee.

The registration scheme of the Royal British Nurses' Association was investigated at some length by the Select Committee of the House of Lords on Metropolitan Hospitals.

From the minutes of the proceedings of the Committee (3rd Rep., p. cxcvi.), it appears that a paragraph was proposed to be inserted in the report to the effect that the Committee considered that the arguments in favour of the registration of nurses outweighed those against it and recommending that a charter should be granted to the Association, but was rejected by their lordships by a majority of six to two. It was pointed out, in the course of the present hearing, that this rejection meant only that the Committee of the House of Lords declined to express any opinion on the question of granting a charter.

The report as ultimately issued contained the following important paragraphs dealing with this subject:—

"An important question affecting the general position of nurses was brought forward in connection with the scheme proposed by the British Nurses' Association, for establishing a general register of nurses. A very broad division of opinion exists regarding the merits of that Association. Its objects, as stated by its advocates, are, 'first, to unite trained nurses together in a purely professional union; secondly, to provide for the local registration of nurses under the control of medical men; thirdly, to help nurses in times of need or adversity; and, fourthly, to improve the knowledge and usefulness of nurses throughout the empire;' and its scheme is declared to be put forth 'in conformity with a great public want and a widespread professional demand.' This statement is traversed in a memorial which was signed by many members of the medical and nursing staffs, and of the governing bodies of hospitals and institutions for the sick in London and the provinces, and which was claimed to represent the majority of those who know most about nursing in this country. The memorial declares that the proposal if carried out 'would lower the position of the best trained nurses, be detrimental to the advancement of the teaching of nursing, be disadvantageous to the public, and be injurious to the medical practitioner.' A petition against the scheme, also largely signed, was presented to the Board of Trade."

"The view taken by the promoters of the Association appears to be that the time has come when nursing should be constituted and legally recognised as a distinct profession, with a central controlling body of its own; in short, that the nursing profession should be governed on much the same lines as the medical profession. The nurses' register would resemble the medical register, and the General Nursing Council would take cognisance of the conduct of all nurses, and would have the same power to strike their names off the register for misconduct, as in the case of the medical profession is exercisable by the General Medical Council. The ultimate object appears to be (whether or not this could be carried into effect at once) to obtain statutory power to prevent any public or private institution sending out women to nurse the sick, who were not registered by a registration board, composed of medical men and hospital matrons, or at all events to prevent unregistered women calling themselves trained nurses. But whether or not there were any such express prohibition, it was thought that a registration board constituted under Royal Charter or Act of Parliament, would have such prestige that the public would decline to employ unregistered nurses. It was claimed that some of the hospitals and many medical officers of hospitals were in favour of registration. The immediate advantage which the public would gain from it was said to be that a reference to the register would at once show whether a woman was a trained nurse or not, and whether she was known to have ever done anything rendering her unworthy of employment, because the name of a nurse would, on sufficient cause shown, be removed from the register; the witness further said, 'it was a very common fraud to steal or forge a hospital certificate.' No hospital is responsible for a nurse once she has left the hospital service; but a General Nursing Council or Registration Board would be responsible to the general body of nurses and to the public; to prevent any woman who proved herself unworthy of trust going on with the work, they would take her name off the register.

"The main point alleged against the British Nurses' Association by its opponents is that it places good and bad nurses on a level. It is urged that neither the completion of a certain period of training nor the passing of a theoretical examination is sufficient guide to the practical fitness of a woman for a nurse's work. Only the institution which has actually trained the nurse, and in which her qualities are recorded after long personal observation, can be in a position to give such a guarantee of her capacity as will be of any practical value. If, for example, a member of the public goes to such a general register for a nurse, he gets someone who has passed through a certain curriculum; if he applies to any nurse-training hospital, he gets a nurse selected for the particular case, and backed by the authority and reputation of the hospital which sends her out. It was further said (in the interests of the medical profession) that the grant of a sort of diploma to nurses might lead many people to seek a nurse in case of illness and not a doctor; such a result, it was thought, would be injurious also to the interests of the nurses themselves.

"Under the existing system it is argued that the public have adequate protection in their power to call for a nurse's certificate before employing her, and to obtain particulars from the hospital which gave it her; that this security would under the registration scheme be lost, and that women, whom no hospital would recommend, would get themselves regis tered and appear to the public on the same level as the best nurses. It was suggested that an official list (if it were needed) could be compiled giving the names of all nurses on the books of the several training hospitals.

"A point very strongly urged is that the character of the woman herself is a most essential matter in regard to a nurse; much more so in the case of a nurse than of a doctor. The Association professes to require evidence of character (by the production of recent testimonials) before it will put a nurse on its register, and to register only women who had had three years' hospital training, but it appears that women are registered who have not completed their full period of training at any one hospital, and of whom it is not known whether they have proved themselves competent or otherwise. The Association complains that a hospital certificate, once given, cannot be withdrawn, whereas a name will be removed from the register whenever a nurse is proved to have forfeited her good character, legal proof being admittedly exceedingly difficult. But it is evident that this course cannot be taken except on clear proof of actual crime or misconduct, and therefore it is no protection to the public from mere incompetency. It was admitted that a woman might go through three years' training at a hospital and get her certificate, and yet be a very indifferent nurse, and be known at the hospital to be so; but the public, who read her name in the register, would suppose her to be competent unless the register clearly stated that it did not guarantee the efficiency of its nurses On the other hand, if the Association disclaims responsibility for the efficiency of the nurses whom it registers, it seems difficult to understand wherein lies the security which it offers to the public.

"Mr. Rathbone, speaking on behalf of the Nightingale Training School in opposition to the Association, quoted from a letter written by Miss Nightingale on this subject: 'You cannot select the good from the inferior nurses by any test or system of examination. But most of all, and first of all, must their moral qualifications be made to stand preeminent in estimation. All this can only be secured by the current supervision, tests, or examinations, which they receive in their training school or hospital, not by any examination from "a foreign body" like that proposed by the British Nurses' Association. Indeed, those who came off best in such would probably be the ready and forward, not the best nurses.'"

### The Association's Petition.

The present application arose upon a petition to Her Majesty in Council, presented by H.R.H. Princess Christian, praying that Her Majesty might be pleased to grant a charter of incorporation to the Princess and the members of the Association, under the name of "The Royal British Nurses' Association."

The petition alleged that the Association was not established for the purpose of gain, but for the purposes of the mprovement of the profession of nurses and of the promotion of their efficiency, and usefulness and of assisting them by various benevolent schemes; and that in furtherance of such objects a list of nurses had been compiled and published, setting forth the names and addresses of nurses, with the names of the hospitals or other places at which they had been trained, and the length of training which each had received, thus enabling the public to form a more accurate judgment of the professional education and experience of the nurses registered. It further alleged that a benevolent fund had been established for the purpose of giving aid to nurses, and that the management of the Association was vested in certain eminent members of the medical profession, matrons of hospitals, and other persons interested in nursing.

### The Draft Charter.

A draft of the charter sought to be obtained accompanied the petition. The purposes of the proposed corporation were there stated to be:—

"1. The maintenance of a list or register of nurses showing as to each nurse registered her name and address, and the names of the hospitals or other places at which she has been trained, and the length of training which she has received.

"2. The founding and maintenance of schemes for the benefit of nurses in the practice of their profession, and in times of adversity, sickness, and old age.

"3. The promotion of conferences and public meetings, and lectures in connection with the general work of the corporation.

"4. The doing anything incidental or conducive to carrying into effect the foregoing purposes."

The draft charter provided that there should be a president and honorary officers of the Association, and that H.R.H. Princess Christian should be the first president, and that the first honorary officers should be the existing honorary officers of the Association, future presidents and honorary officers to be elected by the general council on the nomination of the executive committee. The members of the Association were to consist of the president, the existing members of the Royal British Nurses' Association, and medical men and nurses to be subsequently elected, but, as regards nurses, subject to such conditions as might be prescribed by the bye-laws for the time being in force. The corporation was also to be empowered to provide by bye-laws for the admission of honorary members. Full power to make bye-laws was to be conferred upon the members of the corporation in general meeting, with power to impose penalties.

The government of the corporation was by the charter proposed to be vested in a general council, which was to have power to expel and suspend members, and to elect an executive committee. To the executive committee was to be entrusted the election, in such manner as might be prescribed by the bye-laws, of future members of the corporation. It was also to have power to appoint sub-committees, and delegate any of its duties. It was further provided that the first general council and executive committee should consist of the existing general council and executive committee of the Association, and that they should subsequently be composed of such ex-officio and elected members, and holding office for such term as might be prescribed by the bye-laws.

It is to be observed that the Royal British Nurses' Association proposed to take no power to form a register of midwives, and that the draft charter now sought to be obtained contained no provision as to the character of the list or register of nurses which it was proposed to establish, or as to the machinery by which the register was to be carried on.

A petition in support of the application of the Royal British Nurses' Association was also presented, signed by more than 6,700 persons, including 1,100 medical men, more than 1,000 nurses, 250 clergymen, and a number of other persons.

### Petitions in Opposition.

Petitions in opposition to the application were presented by the Council of the Nightingale Fund, and on behalf of, or representative, of the governing authorities, the medical staffs (or the members thereof specially engaged in instructing nurses) of, and the matrons, lady superintendents, sisters, and other authorities of the nurse-training schools attached to the following large London hospitals, namely, St. Thomas's Hospital, the London Hospital, Westminster Hospital, King's College Hospital, St. Mary's Hospital, Charing Cross Hospital, the Seamen's Hospital Greenwich, and Guy's Hospital (with the exception of the matron), and the St. Marylebone, Paddington, St. Pancras, and Whitechapel parochial infir.

maries, and also on behalf of or representative of the governing authority and medical instructors of nurses of the Royal Free Hospital, and by certain of the medical instructors of nurses of St. Bartholomew's Hospital. Similar petitions were presented on behalf of or representative of the governing authorities, medical instructors of nurses, and training schools of the most important hospitals in Edinburgh, Glasgow, Dublin, Leeds, Liverpool, Manchester, Bristol, and other provincial towns, and of many smaller general hospitals and special hospitals both in London and the country. Similar petitions were also presented by the Metropolitan and National Association for Nursing the Sick Poor in their own Homes and by the East London Nursing Society, and the Liverpool District Nursing Society, and many other institutions in London, the provinces, Scotland, and Ireland for providing trained nurses for the sick poor. One of the petitions was also signed by Miss Nightingale, and others of them by numerous independent persons interested in the welfare of nurses and the advancement of nursing.

The opposing petitions were all in substantially the same form. They alleged that such a list or register of nurses as was contemplated by the draft charter would, if established, be regarded and used by the public as an authoritative guide in the selection of nurses, and that registration therein would be considered a sufficient and conclusive guarantee of competence, and that the Association, if incorporated, would be enabled, by means of the position and dignity accruing from the possession of a charter, and by reason of the control they would have over the register of nurses, to acquire a real power of determining the qualifications requisite to constitute a trained nurse, and of regulating and controlling the training and education of the whole body of nurses. The petitioners submitted that such a register could not be made to supply the kind of information essential in the case of nurses for the sick, such as whether she possessed gentleness, tact, presence of mind, and the other personal and moral characteristics, without which no training could produce a good nurse; that the establishment of the register would be detrimental not only to the general body of nurses, but also to the public and the medical profession; that nurse training was in process of development, and until there was a general concurrence of opinion as to what should be deemed an adequate training, it would be premature and injurious to the progress of nursing to establish a central body with exceptional controlling powers; that numbers of insufficiently trained and inferior nurses would find places in the register side by side with, and (in the eyes of the public) on the same footing as their highly-trained and thoroughly competent sisters; that the register would therefore be untrustworthy and afford no protection either to the public or to medical men, while at the same time an injury would be inflicted on the best nurses, the general standard of nursing would be lowered, and there would be great danger that the incentives to advancement and improvement at present existing might be seriously impaired. It was added that the untrustworthiness of the register would be increased by the extreme difficulty of adequately revising it and of removing the name of any person except for gross misconduct; and that having regard to the existing hospital registers the proposed register was unnecessary.

It was further submitted by the petitions in opposition that even were it desirable that a corporation should be established for the purpose of maintaining a general register of nurses and acquiring controlling power over their education and training, such corporation should be representative of, and should receive the concurrence and support of (1) the general body of nurses, (2) the training schools, and (3) the governing bodies of, at all events, the leading hospitals in the wards of which the training must necessarily be carried on; and that the Royal British Nurses' Association was not representative of any of those classes of persons, and had not up to the present achieved such an amount of success or received such extensive support as entitled it to claim exceptional recognition.

It was, therefore, prayed that the charter might not be granted, and that counsel might be heard and evidence (if necessary) produced in opposition to the application of the Royal British Nurses' Association.

### The Reply.

A detailed reply to the petitions against the grant of the charter was, by direction of the Privy Council, sent on behalf of the Royal British Nurses' Association, in which the objections put forward in opposition and the replies of the Association were set out in parallel columns.

Intimation was subsequently given by the Privy Council that counsel would be heard for and against the application for the charter, and printed statements of the cases of the Royal British Nurses' Association and their opponents respectively were directed to be lodged at the Privy Council office.

### The Association's Case.

The case of the Royal British Nurses' Association stated the reasons for which the charter was sought to be obtained as follows:—

"I. Because the want of an authoritative general register of trained nurses is a public danger, and detrimental to the medical profession, the nursing profession, and those who are in need of nursing aid.

"II. Because the Association now attempts by its register to provide the public with some ready means of ascertaining if any given nurse has received technical education, and in that case where and when she was trained.

"III. Because without incorporation this work of the Association will be impeded, and its value to nurses and the public greatly diminished, while the grant of a Royal charter, conferring the rights and privileges of incorporation, will greatly facilitate the general work of the Association and the carrying out of its benevolent and other schemes.

"IV. Because the principle of an authoritative general register for persons engaged in medical work of any kind has been repeatedly sanctioned by Parliament, and shown to be of the greatest public and professional benefit.

"V. Because when nurses have finished their education and leave the hospital service and work in private houses, they ought to have power effectively to combine for their mutual support and help in times of adversity, sickness, and old age; and the Association, if incorporated, will effectively provide the means of enabling this to be carried out.

"VI. Because the Association is only engaged in a voluntary work, and neither seeks nor desires compulsory powers, or controlling powers over other institutions. It has never interfered, and if incorporated will not interfere, with hospital authorities, who will, if the charter of incorporation is granted, continue to be responsible to the public for the training given to their nurses.

"VII. Because the arguments in the opposing petitions are founded on inaccurate statements, and are entirely answered by the reply lodged on behalf of the petitioners.

"VIII. Because the work of the Association is of the greatest public benefit and importance, and for the public welfare a Royal charter ought to be granted to it."

In the body of the case stress was laid on the system of voluntary registration which had been in force for two years. It stated: "Careful inquiries are then made by the Registration Board as to the truth of the applicant's statements as to her moral character. If these are satisfactory, the nurse's name, address, and when, where, and time when she was trained as a nurse are entered on the register. The Registration Board have power to remove from the register the name of any nurse who proves herself to be unworthy of trust."

In paragraph 12 of the case the Association submitted the following statements:—

"(1) The present state of affairs in the nursing profession is most dangerous to the public, detrimental to medical treatment, and discreditable to nurses. This is shown by the facts that:—

"(a) Any woman, however destitute of knowledge or of character, or of both, can term herself a trained nurse, and can obtain employment in that capacity.

"(b) Certificated nurses, who prove themselves unworthy of trust, by drunkenness, theft, or by commission of even graver offences, can continue to practice their profession, under cover of their original certificate of efficiency. There are at least 200 hospitals in the United Kingdom at which women are trained as nurses, and certificates of competency are given by the authority of the majority of these hospitals. Very few of these authorities keep any detailed record of the women whom they thus present to the public as skilled workers. When these women leave the hospital service it is manifest that their former employers cease to have any authority or control over them, and have no power to cancel or recall any certificate which they have awarded, even if the possessor may subsequently prove herself to be utterly unworthy of trust. Therefore, although hospitals may dismiss for grave faults nurses whom they have previously certificated, these nurses can afterwards claim to be trustworthy on the authority of the very institution which has been compelled to discard them."

The Case in Opposition.

The case in opposition submitted the following reasons as those which should prevent the charter being granted:—

"1. That a general register is not adapted to the calling of nurses for the sick, and that any possible register of nurses would be misleading to the public and detrimental to the interests of nursing.

"2. That the proposed register of nurses is in no way analogous to the existing register of medical men, and that the arguments in support of the latter do not apply to the former.

"3. That a register of nurses could not be effectually carried on except under statutory powers.

"4. That any attempt to maintain such a register under the authority of a charter would lead to mischievous results.

"5. That the grant of a charter would enable the Royal British Nurses' Association to acquire a real if indirect power of controlling the education of the whole nursing profession.

"6. That the Royal British Nurses' Association is not a sufficiently representative body, and that it has not secured sufficient support or achieved sufficient success to entitle it to be entrusted with such powers.

"7. That it has not the means of discharging the duties and responsibilities which the charter would impose upon it.

"8. That the establishment at the present time of any register of nurses would be premature and injurious.

"9. That a formal register of nurses is unnecessary.

"10. That the other objects for which incorporation is sought can be accomplished without the grant of a Royal charter."

The body of the case went through the history of the improvement in the training of nurses which has taken place during the last thirty years, and insisted on the primary importance in the case of a nurse of the moral and personal characteristics which have been already referred to. It then set forth an outline of the nature of the training given by, and the system of training adopted in the most advanced training schools. It also pointed out that besides the larger hospitals, there were a great number of smaller general and of special hospitals which profess to take probationer nurses, and give them some kind of training.

Paragraph 20 was as follows:—

"Thus the certificates at present granted to nurses by different hospitals differ most widely in value; nor does any general uniformity of system in the training of nurses prevail, nor is there any general concurrence of opinion either as to what constitutes an adequate training or what should be the minimum qualification for a hospital undertaking to train. Much larger experience than has at present been available and a very considerable development in the means of training are necessary before any such general uniformity of system or opinion can be looked for."

The case proceeded to state the history of the movement in favour of registration and of the efforts to that end which had been made by the Royal British Nurses' Association, and it was submitted that the object of the Association had throughout been to establish an authoritative register by which the skill and competence of a nurse might be certified to the public.

By paragraph 37 it was submitted that the establishment of a general register was not adapted to the calling of nurses for the sick, because (among other reasons) it could not disclose nor could any registering authority adequately test the existence of the present moral characteristics already maintained, and that, therefore, it could not fail to be untrustworthy and misleading to the persons relying on it. The paragraph then proceeded:—

"At the same time, by reason of the fact that the register would inevitably contain the names of numbers of insufficiently trained nurses, side by side with, and (in the eyes of the public) on the same footing as their highly trained and thoroughly competent sisters, an injury would be inflicted on the best trained and most competent nurses, the general standard of nursing would be lowered, and there would be great danger that the incentives to advancement and improvement, which at present exist, would be seriously injured."

The case further stated that it was believed that a register of trained nurses, professing to be an authoritative register, by which the skill and competence of a nurse should be cerfied and put forward under the authority of a chartered corporation, would come to be regarded and used by the public and by medical men as an authoritative guide in the selection of nurses, and that registration therein would come to be generally accepted as a sufficient and conclusive guarantee of competence. It was pointed out that the formation and maintenance of such a register would involve, in the case of applicants not possessing or unable to produce a hospital certificate, the whole duty of sifting the alleged qualifications for registration, and in other cases the duty (at least) of ascertaining the genuineness of the certificate produced, and of investigating the subsequent history of the applicant. It was accordingly submitted that the register could not be successfully carried on unless—(1) The registering authority had power to control both the training schools and the hospitals; (2) it were rendered compulsory on training schools, hospitals, and other institutions and persons to furnish the registering authority, with all requisite information in their possession; (3) penalties were imposed for fraud and for the falsification of the register; and (4) not only the duty of the registering authority, but also the rights of the persons seeking registration were defined and established.

Addresses by Counsel.

Sir Horace Davey, in opening the case on behalf of the petitioner, said the Royal British Nurses' Association was formed about four years ago for the following objects: (1) To protect the public, the medical profession, and trained nurses by establishing a system of registration for trained nurses, similar to that which had been established by Parliament for medical men; (2) to form and maintain schemes for the benefit of nurses in the practice of their profession, and in times of adversity, sickness, and old age; and (3) other objects beneficial to the profession of trained nurses and those who require their services. Soon after its formation the Association applied to the General Medical Council to undertake the registration of nurses. The council, however, while approving the system, declared itself unable to carry it out. Next, the Association applied to the authorities of all the large hospitals to form a body for the purpose mentioned, the registration of trained nurses, but their application was not acceded to. The Association then appointed a registration board, and initiated a system of voluntary registration of trained nurses, which had been in operation for two years. Besides this the Association had carried on important schemes for the benefit of its members, and in 1890 it learned that large sums of money might be entrusted to it, to be administered for the benefit of trained nurses, if th e

Association could be incorporated. The Board of Trade was applied to for a licence to permit the registration of the Association under the 23rd Section of the Companies Act, 1867. This was refused on account of objections made, the nature of which was not disclosed. Afterwards the objections were supplied to the Association, and they contended, and were prepared to prove, that the statements were inaccurate and misleading. The Board of Trade was unable to investigate the matters in dispute, but offered to support an application to the Privy Council for an inquiry. The proposed corporation asked (1) to be empowered to maintain a list or register of nurses, showing where they had been trained, and the length of such training; (2) to found and maintain schemes for the benefit of the nurses; (3) to be allowed to promote conferences, public meetings, and lectures; and (4) to do anything inciden tal or conducive to carrying into effect the foregoing purposes. The Association numbered 2,821 members, and had spent in two years a sum of not less than £800 on the work of voluntary registration. A petition in favour of it was presented, signed by more than 6,700 persons, including 1,100 medical men living in every part of the United Kingdom, more than 1,000 nurses, 250 beneficed clergymen, and a considerable number of persons eminent in the professions of the army, navy, law, and science. The petitions in opposition contained very important and weighty signatures, which he did not desire to underestimate. Among others was the respected name of Miss Nightingale; but with regard to her, he must point out that she had for years, owing to regrettable circumstances, been unable to take any active part in nurse training. The great object which the Association had in view in proposing to establish a register was to save the public from inefficient nurses. Any person who received a certificate from the hospitals or training-schools might go out into the world and obtain employment, whatever her character or experience might be. The consequence was that many persons obtained employment as nurses who were unfit by character, or, by want of experience, were incompetent. It was proposed by the Association to establish a register of nurses, which should be founded on the reports of the hospitals and training schools where they were trained, and also upon references as to the conduct and character of the person seeking registration. More it could not do. The Association did not intend to train or do more than inquire into the competence and the moral character of the person applying for registration. That was the first object the society had in view. The registration was purely voluntary, and in no sense coercive. There were 2,700 nurses on the register of the Association.

Sir Richard Webster said the number was 1,700.

Sir Horace Davey replied that his instructions were that there were 2,700 registered nurses at the present moment. Considering that it had had the opposition of some of the hospitals and training schools, the Association had had a large measure of success. A great deal of the opposition to the Association was founded on misunderstanding. He wished to make it perfectly plain that the Association in no sense undertook the training of nurses, but left that entirely to the useful institutions at present engaged in it. The Association did not in any sense whatever train nurses. It did not pretend to train them. It had no machinery for the purpose. It left that entirely in the hands of the hospitals and training schools, and did not desire to dictate to them in any way with regard to the work in which they were engaged. The action of the Association was supplemental and auxiliary to the hospitals and training schools, and not in opposition to them. The register would not be authoritative or final as to the qualifications of a nurse. All the petitioners sought to do was to establish a register from which the public when they wanted a nurse would see where she had been trained and during what period. It would give no control over nurses or the training schools.

Lord Hobhouse: Would you infringe your charter if you went beyond that?

Sir Horace Davey said the Association would be prepared to put on the register any person who possessed the required qualification and asked to be registered. The work was intended to be entirely voluntary, and any weight the register might have would depend on the value the public might attach to it. With regard to revision of the register, they would endeavour to keep as far as possible in touch with the nurses, but he admitted that it would be impossible practically to carry out any complete periodical revision.

The list of members of the registration board of the Association contained the names of members of the medical profession of great eminence. The regulations for registration required that applicants for registration should have been engaged in work for three years in hospitals or infirmaries. The list of the members of the Association whom it was proposed to incorporate contained the names of medical men of the highest eminence and of ladies engaged in the profession of nursing not only in London, but in all parts of the United Kingdom. In opposition to the incorporation there were 280 medical men, 540 nurses, and 140 of the general public. Their lordships would find that, almost without exception, the opposition proceeded from some of the training schools and some of the hospitals who trained nurses, and who derived a large part of their income from sending out nurses. A great deal of the opposition arose from the regulation which required that a nurse should have had three years' training before she was entitled to be put on the register. Most of the hospitals and schools required a much shorter period of training. The petition was opposed by the council of the Nightingale Fund. Sir Horace Davey referred to the Select Committee of the House of Lords on Metropolitan Hospitals, the report of which committee stated that nurses should have three years' training, and said that that was why three years' training was required by the regulations of the Association. The petition for the incorporation of the Association was supported by St. Bartholomew's Hospital, St. George's Hospital, Middlesex Hospital, and University College Hospital. On the other hand, it was opposed by St. Thomas's Hospital, King's College Hospital, the London Hospital, and many other hospitals. It was objected that the Association desired to regulate and control the training and education of the whole body of nurses. It desired nothing of the kind. Another objection was that a register could not be made to supply the kind of information which was essentially required in the case of nurses for the sick, such as the possession of gentleness, tact, presence of mind, and other personal and moral characteristics, without which no training could produce a good nurse. Perfectly true. Neither their list nor any list in the world could do that. Information as to that could only be obtained by personal acquaintance. But, because it could not do that, did it follow that a list would be of no value? Let the training schools publish their registers. The essence of the Association's case was that there were no registers of nurses accessible to the puplic. Then it was said that a register such as was contemplated by the draft charter would be detrimental, not only to the general body of nurses, but also to the public and the medical profession. He asked their Lordships to form their own opinion. Why it was detrimental he did not know. No one denied that it would prevent incompetent and improper persons from being employed, and if they did that they were to be congratulated.

Their Lordships intimated that one counsel only would be heard.

Sir R. Webster addressed the Committee on behalf of those who opposed the granting of the charter. He said he appeared on behalf of a large and influential body. The vast majority of those interested for years in the training of nurses, and the nurses themselves, were opposed to the granting of the charter, especially with regard to the provisions as to a register. Some three or four of the metropolitan hospitals supported the petitioners, but all the rest and nine-tenths of the provincial hospitals concurred in opposing the application. Sir H. Davey had, no doubt unintentionally, somewhat depreciated the great services of Miss Nightingale. That lady, although of late she had been in ill-health, continued to take a most active and energetic interest in the whole question of nursing; and almost every large training school was to this day carried on in accordance with her views.

Lord Hobhouse said he did not think the learned counsel need labour the point as to the weight which ought to be attached to the opposition.

Sir R. Webster proceeded to contend that a general authoritative register of nurses would be seriously detrimental to the nursing profession, and that it would be a very great evil to the public and the medical profession who desired trained nurses if this charter were to be granted. If anything of the kind were to be done, it must be surrounded by such safeguards and controlled by such provisions as were found in the Medical Act, the Pharmacy Acts, and the Veterinary Act, under which the various registers connected with the medical profession were carried on. He believed that to establish a general register by charter was absolutely without precedent. Some of the powers asked for by the present charter, as, for instance, the power to impose penalties, were quite unprecedented. The contention of the Association had throughout been that the register of nurses should be an authoritative record, to which the public could go when they wanted a trained nurse, and see who were, and who were not, competent nurses. It would be wrong and most dangerous to attempt to establish such a register in any way except by Act of Parliament. The difficulty of establishing such a register was, in fact, fully admitted by the petitioners. The learned counsel then read the paragraphs from the Lords' Committee on Metropolitan Hospitals printed above, and referred to the refusal of the Committee to advise the grant of a charter to the Association, and proceeded: It was said that an imperfect register would be better than no register. He denied that. No register could give information as to the possession of tact, gentleness, presence of mind, and other personal and moral characteristics. It would therefore be misleading, and if misleading injurious. This register would not be of equal value to that which was kept at the various training hospitals for nurses where the character of the nurse was best known; yet it was designed to supersede all other lists and be the authoritative list.

Lord Hannen said that the register would only show people where they could go and get special information.

Sir R. Webster urged that the public could not distinguish between the different qualifications appearing on the register. It would look only at the fact of registration and take that as authoritative.

Lord Hannen observed that that would be the fault of the public.

Lord Hobhouse: Your point is, is it not, that the register might put people off inquiry?

Sir R. Webster said that was so. The list would be regarded as an authentic certificate, and that would open up a serious danger. There were many nurses who passed a creditable examination, yet who, by reason of their temper or habits, were unfitted to become successful nurses.

Lord Hannen: Is it not a useful thing that there should be a place where the public could go and see the qualifications of a nurse?

Sir R. Webster: And a dangerous thing, because the register would not show the essential qualifications of a nurse, her tact, her disposition, her temper, and presence of mind. Unless it was compulsory for every nurse to be upon the register it would be misleading.

Lord Oxenbridge: Those who wanted to be engaged would get upon the list.

Sir R. Webster insisted that, instead of being a useful directory, the register would be detrimental to the training of nurses and to their reputation as nurses. It would also be a disadvantage to the patients themselves. The register could not be regarded as other than a certificate of merit.

Lord Hannen: If a small-pox nurse was wanted inquiry for the special nurse would be made. If a nurse for any special object was desired that kind of nurse would be asked for.

Sir R. Webster said that the tendency of the register would be to put the paper qualification higher than the actual qualification. The register also would be of no value unless it was compulsory. The register was intended to prevent the employment of incompetent nurses. If it was to do that it must be something more than a mere record of hospital certificates. The views which he was putting forward were those entertained by a great body of medical men and nearly all the training schools.

The learned counsel then proceeded to read extracts, showing the opinions upon this point of Miss Nightingale, Mr. Bonham-Carter, the Secretary of the Nightingale Fund, and Miss Lückes, the Matron of the London Hospital. He had not finished his arguments when the Committee rose.

The case was adjourned to Monday, the 28th inst.

### Appointments.

Parish of Christ Church, Battersea.—Nurse Jane Rogers, who trained at Charing Cross Hospital, and was at St. John's House over five years, has been engaged as nurse to this parish. Nurse Rogers passed her L.O.S. examination from St. John's Maternity House, Battersea. We are especially glad to hear of the appointment, as it will lessen the gap left by the closing of St. John's Maternity Nursing Home.

Lady Strangford Hospital, Port Said.—Miss Henrietta Hamilton, who was for three years Matron of the Princess Alice Memorial Hospital, Eastbourne, sailed last Thursday in the "Mirzapore" to take up the Matronship of the Lady Strongford Hospital, Port Said. Miss Hamilton trained at St. Bartholomew's Hospital, and in 1881 became Sister of "Sophia" ward at the London Hospital, which post she held many years. Since leaving Eastbourne last June she has been taking holiday duty for matrons in different institutions.

North Lonsdale Hospital.—Miss M. L. Boulton, who has been for some time the sister in charge of the Nicholson Ward of the Bolton Infirmary, has been appointed Matron of the North Lonsdale Hospital at Barrow-in-Furness. Miss Boulton received her training at the Edinburgh Royal Infirmary, and subsequently officiated as a "sister" at the Southern Hospital, Manchester, from which institution she came to the Bolton Infirmary, throughout her connection with which she has retained the confidence of the committee and the medical staff, and the esteem of the patients who have been entrusted to her care.

Clayton Hospital, Wakefield.—Miss Amy Eaton has been elected Matron of this hospital. The appointment took place on November 9th, and there were sixty-two candidates. Miss Eaton was trained at the Derbyshire Royal Infirmary for three years, and at the end of her training she became head nurse, and held that post for five years, when she left to take up the work of Sister to the adult wards of forty-two beds at the Royal Albert Hospital, Devonport. In June, 1890, she was appointed Night Superintendent at Bristol General Hospital, and a year later she succeeded Miss Cox as Assistant Matron there. Miss Eaton holds excellent testimonials, and is well fitted to undertake a matron's duties.

### Everybody's Opinion.

*[Correspondence on all subjects is invited, but we cannot in any way be responsible for the opinions expressed by our correspondents. No communications can be entertained if the name and address of the correspondent is not given, or unless one side of the paper only be written on.]*

### EXAMINATION PAPERS.

"A Private Nurse" writes: Having found The Hospital a great help in gaining information, may I venture to suggest that the examination questions for nurses would be doubly useful and helpful were the competitors obliged to send in their answers without having first looked up and prepared the subject? Surely, after such preparation, anyone could send correct and practical answers. Could you not, with equal benefit to the majority of your readers, make a rule that the answers should be written from the nurse's own personal knowledge and experience? Most hospital-trained nurses have gone through, and probably passed some examination during their training, when the theoretical part of the work would be taught, though possibly it might be forgotten; but the being obliged to think out these answers would brighten up old knowledge. For my own part, I hope to gain help in this way (though certainly not the prize), but fail to see how I could do so if I first got all my information before writing from some manual.

### NURSES' FEES.

"A Patient" writes: Illness has prevented me from writing sooner, but I must tell you how glad I was to see your wise and thoughtful remarks as to the cost of trained nurses. I have benefited too much by their ministrations not to feel that it is impossible to value their services too highly, or to show them too much gratitude and consideration. But this very appreciation causes one to sympathize very keenly with those of whom £2 2s. per week and the very heavy extras render the services of a trained nurse unattainable, and one cannot help wishing that skilled nursing should be within the reach of that large class which is just above being able to accept charity, but to whom the terrible expense of a serious illness means privation or loss of necessary comfort for many a day. I should like to assure you that you will have at least one appreciative reader, who trusts that you may see your way to advocate some reduction in nurses' fees, especially when the lion's share is taken by the institution acting as middleman.

### NURSING IN BOMBAY.

"Four and a-half Years' Nurse" writes as follows; when our readers can give definite information to their fellow nurses we shall be very glad: Will you kindly let me ask in your journal if anyone can tell me anything they know of the European Hospital, Bombay, and how to get an apppointment there; also if there is an infectious hospital there? I should be greatly obliged,

### Notes and Queries.

**Queries.**

(31) *St. Mary's, Plaistow.*—Will any midwife trained at St. Mary's Home, Plaistow, give particulars of expenses, uniform required, the cost of washing and extras and oblige a nurse of limited means.—*H. S.*

**Answers.**

(31) *St. Mary's, Plaistow (H. S.)*—The charge at St. Mary'sHome for training in midwifery is 13 weeks 13 guineas; six months, £20. You would wear a washing dress, preferably of blne grey skirting, white apron with bib and straps, plain black bonnet, and plain black circular cloak. You probably have all these, and caps are provided at the home, 1s. 6d. would be the very least for washing, it would often be a little more, perhaps. Any more information perhaps a reader will give you.

### Wants and Workers.

*Wants at Weymouth.*—The Matron of the Weymouth Royal Hospital would be glad to receive a musical box as a Christmas gift for the use of the patients. Also old linen would be most acceptable.

*Surgical Instruments.*—The Matron of St. Mary's Cottage Hospital, Plaistow, E., would be very glad of second-hand surgeon's instruments. The hospital is a new one and much in need of help.

*A Home for an Epileptic.*—Can anyone tell me of a permanent home for epileptic patients where a girl of eleven, subject to epileptic fits could be received for a small weekly payment? Not eligible for Hospital.—*M. E. P.*

### Notes on Novelties.

### THE SANITARY WOOD WOOL COMPANY.

We have received from Messrs. Hartmann a "guinea" box containing the necessary appliances for use in confinement cases. We are glad to be able to say that we find every requirement has been considered, and that the absorbent antiseptic sheets and towels are soft, yet substantial, and of excellent sizes. Besides a macintosh sheet and three wool sheets the box contains four pockets of towels (some larger, some smaller), a strong linen binder, a box of oiled silk, a box of Fuller's earth, carbolised vaseline, pins, thread, and two vaccination pads. A few simple directions are also enclosed. We consider this "guinea" outfit very moderate in price, and the hygienic benefit of such an equipment is so well known that it needs no praise from us. All Messrs. Hartmann's manufactures may be obtained from their "Manageress," 26, Thavies Inn, Holborn Circus.

### SHETLAND HAND-KNIT GOODS.

The vests and other garments made by Mr. John White, of Edinburgh, of the pure Shetland wool would, we imagine, convert the most hardened opponent of woollen underwear, for it is light and elastic, and yet warm enough to keep out the extreme cold; in fact, it is the nicest form of woollen clothing we have yet met with. Vests are made in all sizes, from the smallest infant upwards; and, speaking of babies, many of them, we feel assured, would suffer many less discomforts if they were dressed in these Shetland vests. We have visions of small arms being forced into hard, tight garments, and by the use of elastic loosely knitted things all this tugging is obviated; for the same reason invalids would find this underwear quite invaluable. Spencers and jackets, shawls, gloves, mittens, socks, and stockings can all be obtained from the warehouse in Frederick Street; and homespun tweeds, made by men on the old-fashioned hand-looms, are also kept. But the best plan is to write for a catalogue, as space will not permit us to give a very lengthy category of its contents.

### Short Items.

We acknowledge with many thanks the receipt of half-a-crown for the endowed bed for a sick nurse from Nurse Annie.—The response to the appeal of the Ladies' Committee for funds to supply a district nurse in St. Ives has been such as to enable the Committee to engage a nurse, who is to commence work some time in the course of next month.—In connection with Colonel Gildea's scheme for providing trained nurses for the assistance of soldiers' and sailors' families in garrison towns, a nurse has been appointed at York and in the Curragh, so that the scheme seems to be on the road to rapid development.—At the annual general meeting of the East Mill Company (Limited) held at Brechin recently, it was resolved to give a donation of seven guineas to the Brechin Infirmary, and one of three guineas to the branch of the Victoria Nursing Institute which is being established at Brechin.—Arrangements have been made in Dublin for the various district nurses to attend soldiers' families in the St. Patrick's Home, under orders from the chief army medical officer of the district.—A legacy of £100 from the executors of the late T. Carter, Esq., has been received by the treasurer of the Norfolk and Norwich Staff of Nurses.—Lady Helen Munro Ferguson opened a sale of work in aid of the Edinburgh branch of the Q.V.J.I.N. last week, held at the Nurses' Home, Edinburgh.—Westbury is to have a parish nurse.—The work of Nurse Pike, who for six months has been working single-handed at Taunton, has been so successful that a maternity nurse will probably be engaged to extend the work still further.

### The Muses' Looking-glass.

LA CITÉ DE MISÈRE.*

Under this rather ill-judged title M. Milès presents his readers with a picture of hospital life from an outsider's, and what might be called an impressionist's, point of view. Common sense, aided by the continual pressure of hard work, has greatly contributed to exclude any touch of the sentimental from English hospital administration. And it is well that it should be so, for disease and death in close contact are best met with entire simplicity and reverent reticence. This effort to evoke complex emotions from the contemplation of suffering has not even the excuse of serving to rouse public generosity, since the Parisian hospitals are maintained by the State. But in any case it must be doubted whether the shudder of horror cheaply raised by a visit to the operating and dissecting rooms is likely to be productive of substantial sympathy.

But leaving on one side the mixed æsthetic and realistic element which pervades the book, there is much to interest in this detailed account of the oldest Parisian hospital. The Hospital of St. Louis, built in 1603, may well claim the title of City. Originally intended for plague patients, it lay far outside the old limits of Paris, and was situated in a vast extent of well-wooded ground, enclosed by walls flanked with turrets. Within these walls lie now two distinct sets of buildings known as the old and the new town. In strange contrast with the massive architecture, high windows, and sombre cloisters of the old building are the newly-constructed baths, medical schools, consulting rooms, and other modern appliances of science. Here is the studio where a skilled artist, M. Barretta, dignified with the Legion of Honour, sketches from the life new developments of disease, and prepares diagrams for the student. A little further on the photographer is busy with cases not deemed of sufficient importance for the art of M. Barretta. Another studio is devoted to the preparation of skeletons and subjects for the museum. Quite a world of industry is here. In this shop shrouds of various kinds may be purchased. Close by the manufacture of coffins is proceeding, and less ominous industries, carpentering, baking, tailoring, &c., are in full swing in workshops of every description, for everything needed in the working of the hospital is produced on the spot, and gasworks and a tall factory chimney lie in close proximity to the wards. M. Milès is strongly of opinion that in cases where the general health is only slightly affected by local disease, the patients might properly undertake much of this work. It has only, however, so far been found practicable to employ female patients with needlework. Incurable female patients are frequently retained under treatment and admitted to work in the laundry for wages, and thus a branch of refuge work is added to the hospital. But the functions of St. Louis do not stop there. So many little patients there were, suffering from the familiar troubles English mothers class together as "a bad head," that a regular school has been formed for them "école des épilés"—to which outsiders also are admitted. By this means the long isolation and stoppage of education which "ring-worm" entails on its small victims is avoided, and by the researches of Dr. Lallier, who founded and directs the school, the period of treatment has been not only enlivened, but considerably shortened.

The regular hospital affords accommodation for 1,200 patients. Of these the majority are suffering from various forms of skin disease, which forms the speciality of the hospital, but a few wards are reserved for general cases, accidents, and fever. The treatment of patients after operations presents many novel features.

"For those who have undergone any great operation little pavilions or huts have been built at the back of the hospital. Each pavilion contains one or at the most two beds. A wide opening at the side affords a free circulation of air. Outside, near the threshold, a kind of porch shelters the nurse, and contains a stove which regulates the temperature. And all round are the big trees, the grass and flowers, with no street noises. Sometimes a hen comes in at the open door to the foot of the patient's bed in search of grain or insect; sometimes a rabbit scuttles by timidly, or a cat walks in with majestic familiarity, and the patient's over-excited feelings slowly subside into a placid languor in harmony with his surroundings. At night the song of the birds, the view of the far-off hills, and the distant chiming of the Angelus dispose him for sleep, while the Sister of Mercy tells her beads at his side, and nothing reminds him of past suffering. And in the peace which passes from the body to the soul the work of healing is accomplished more patiently, more surely."

This is a wonderful break out of red-tapeism. Unhappily the evils of officialism are not always avoided. M. Milès justly reprobates the extraordinary want of taste which affixes to each bed a form containing among other details respecting the patient, blank spaces labelled "date of death," and "particulars of post-mortem examination." M. Milès' remarks are sensible, if slightly sensational: "It does not kill you to see the words post-mortem examination on a paper which bears your name, but there is, at any rate, nothing very reassuring about it. Who knows if in the long silence of the night a feverish patient may not be visited with a sensation of a cold marble slab, a sensation of a fine blade marking out large sections in his organism, and exposing to view the naked wheel-work of his disorganised machine!"

The ordinance which condemns the hospital attendants to a uniform diet of the beef from which the patients' broth has already been made sounds slightly unreasonable, though no doubt French cookery makes many curious things palatable. It is not surprising that the attendants consider they have just cause for complaint.

On the whole, with its zealous and sympathetic band of doctors, with the self-effacing ministrations of the Sœurs de Charité, with the beauty of its surroundings fringed with irises and studded with ancient trees, and with every alleviation which science and mercy can suggest, St. Louis gives the impression of an admirably-organized city of healing and good works. And no doubt the busy workers, and many of the patients themselves, too, would assure M. Milès that there are alas many sadder places in the world than his so-called City of Misery.

* "La Cité de Misère." By L. Roger Milès (Marpon et Flammarion).

### Where to Go.

St. George's Hall.—On Tuesday, November 29th, at eight o'clock, the Strolling Players will give Bronson Howard's "Old Love and the New." The performance is in aid of the Surrey Convalescent Home, and, if a lengthy list of patrons promotes success, funds should roll in. Reserved seats, 7s. 6d., 5s., and 3s.; unreserved, 1s. Tickets may be obtained from the office of the Home, 15, Cockspur Street, S.W.

Sale of Work.—The sale of work in aid of the funds of the Trained Nurses' Club, 12, Buckingham Street, Strand, will be held at 27, Fellows Road, South Hampstead, on November 26th, from three to ten o'clock. It is very much hoped that all interested will do their best to promote its success by going or getting friends to go. There is no entry charge, and tickets may be had from the club. Fellows Road is quite near the Swiss Cottage and Chalk Farm Stations, and is also close to the Adelaide, Haverstock Hill, whence there is a constant service of omnibuses to all parts of London.

### Minor Appointments.

[We propose to insert from time to time under this heading the appointments of Assistant Matrons, Charge and Staff Nurses, if our readers will kindly keep us informed of their promotion.]

London Hospital.—Miss Alice Moreing, who was trained at the London Hospital, has been appointed Sister of Sophia Wards; and Miss Hattie Oleson Luckie, also trained at the London Hospital, has been made Sister of Queen Victoria Wards.

